# Long-term administration of probiotics prevents gastrointestinal mucosal barrier dysfunction in septic mice partly by upregulating the 5-HT degradation pathway

**DOI:** 10.1515/med-2023-0869

**Published:** 2023-12-26

**Authors:** Xiaopeng Cao, Hui Zhao, Zhimin Liang, Yi Cao, Min Min

**Affiliations:** Department of Gastroenterology, The First Medical Center of PLA General Hospital, Beijing, 100048 China; Department of Gastroenterology, The Fifth Medical Center of PLA General Hospital, No. 51 Fucheng Road, Haidian District, Beijing, 100039 China; Department of Global Health, Milken Institute School of Public Health, The George Washington University, Washington DC, 20052 USA

**Keywords:** sepsis, probiotics, gastrointestinal barrier, 5-hydroxytryptamine, ERK signal

## Abstract

Sepsis can impair gastrointestinal (GI) barrier integrity. Oral probiotics (PT) can maintain the balance of GI microflora and improve GI function. 5-Hydroxytryptamine (5-HT) is a key promoter of GI injury caused by sepsis. However, the mechanism by which PT attenuates sepsis by regulating 5-HT is not fully understood. In this study, C57BL6 mice were intragastric administrated with normal saline (NC) or PT once a day for 4 weeks before cecal ligation and puncture (CLP). Compared with NC-CLP mice, PT-CLP mice had lower clinical score, higher body temperature. The survival rate of PT-CLP mice was significantly improved. The levels of inflammatory cytokines and 5-HT were obviously decreased in PT-CLP mice, and GI peristalsis and barrier function were enhanced. Moreover, sepsis downregulated the expression of tight junction proteins, while PT pretreatment could maintain them at the level of sham operation group. Furthermore, PT pretreatment increased the expression of serotonin transporter and monoamine oxidase A. PT administration could inhibit NF-κB activity, and activate ERK activity. In conclusion, long-term supplementation of PT before CLP can prevent sepsis-induced GI mucosal barrier dysfunction in mice, which may be partially mediated by upregulating the 5-HT degradation pathway via activating ERK signaling.

## 1 Introduction

Sepsis is a systemic inflammatory response syndrome caused by the invasion of bacteria and other pathogenic microorganisms, which can be caused by infection in any part of the body [1,2]. Sepsis often occurs in patients with serious diseases, such as severe burns, multiple injuries, postoperative patients, and so on, which has become one of the important causes of death in intensive care unit [3]. In the initial stage of sepsis, inflammatory cells such as macrophages and neutrophils are activated to produce a large number of pro-inflammatory cytokines, such as interleukin-1β (IL-1β), IL-6, and tumor necrosis factor-α (TNF-α), as well as reactive oxygen species lead to endothelial and epithelial damage, affect vascular permeability and heart function, and eventually result in tissue necrosis and organ failure [4]. The stress response caused by serious disorders often destroys the intestinal mucosal barrier, causes intestinal dysfunction, and microflora ecological imbalance, and then leads to the translocation of intestinal bacteria/endotoxin, which is closely related to the subsequent sepsis and multiple organ dysfunction [5].

Regulating intestinal microflora balance by taking probiotics (PT) has been shown to improve intestinal function and alleviate sepsis [6,7]. It is believed that PT can compete with pathogens for binding site and nutrients, and meanwhile can produce bacteriocins to kill pathogens. Yang et al. found that PT such as *Lactobacillus rhamnosus* GG (LGG) can eliminate pathogens, promote mucosal immunity, and resist Salmonella infection [8]. It is reported that LGG may also reduce rotavirus-associated diarrhea by increasing interferon-γ [9]. Moreover, PT can regulate immune response and reduce inflammation through IL-10 and transforming growth factor (TGF-β) signal [10,11].

5-Hydroxytryptamine (5-HT), also known as serotonin, is an important regulator of the gastrointestinal (GI) tract [12]. It has been confirmed that the intestinal microbiota can regulate production of 5-HT from the enterochromaffin cells [13]. During sepsis, dysregulation of 5-HT induced by microflora ecological imbalance may promote inflammation, damage the intestinal mucosal barrier, facilitate bacterial translocation, and increase the mortality of sepsis [14]. Recently, Wang et al. reported that PT intervention can reduce serotonin status [15]. So, can supplementing PT prevent the pathological response caused by sepsis through reducing 5-HT. Moreover, the mechanism by which PT regulate 5-HT is not fully understood. Therefore, in this study, a sepsis model induced by cecal ligation and puncture (CLP) was established in mice pre-fed with PT for 4 weeks to detect the preventive effect of PT supplementation on the GI mucosal barrier and explore how PT pretreatment can improve GI function by regulating the 5-HT pathway.

## 2 Methods

### 2.1 Animal studies

Animal protocols were approved by Committee on Ethics of the Affiliated Hospital of Academy of Military Medical Sciences (ky-2015-6-27). Male C57BL6 mice (4–5 weeks old) were purchased from Shanghai JieSiJie Laboratory Animal Co., Ltd (China) and kept under controlled conditions (room temperature of 23 ± 1°C, 12 h/12 h light/dark cycle, 50% humidity, free access to a sterile diet and water).

The PT with the trade name BIFICO are composed of triple viable microbe: Bifidobacterium, Lactobacillus, and Enterococcus, purchased from SPH Sine Pharmaceutical Laboratories Co. Ltd (China). Long-term oral administration of 200 μL of normal saline (NC-sham group, *n* = 20; NC-CLP group, *n* = 20) or PT (2 × 10^8^ CFU/mL; PT-sham group, *n* = 20; PT-CLP group, *n* = 20) to mice before CLP, once a day for 4 weeks.

The experimental sepsis model of mice was established according to the previous studies [16,17]. Briefly, the mice fasted overnight before CLP but were allowed water *ad libitum*. The mice were anesthetized by intraperitoneal injection of a solution of 1:1 ketamine (75 mg/kg) and xylazine (15 mg/kg) at a dose of 30 μL/20 g body weight. A 1 cm incision was made on the left to midline of abdomen, and the cecum was moved out of peritoneal cavity through the incision carefully. In order to achieve a mid-grade sepsis, the cecum was ligated by 60% and punctured from mesenteric to anti-mesenteric side using a 21G needle. A small amount of feces was squeezed out of the holes and the cecum was put back into the peritoneal cavity. Finally, the abdominal musculature and abdominal skin were closed in layers. The sham mice received the same surgery except the cecum ligation and puncture. After operation, all mice were intraperitoneally injected with 1 mL of prewarmed NC to supplement heat and water lost during the procedure and placed under a heat lamp until recovery. Ten mice in each group were monitored to assess mortality for 120 h.

After 24 h of CLP, body temperature and clinical score were measured according to the literature [18]. When mice were anesthetized, the temperature was measured by inserting a temperature sensor into the rectum. The clinical score was assessed as follows (points): (i) appearance: normal (0), lack of grooming (1), piloerection (2), hunched up (3), above and eyes half closed (4); (ii) behavior-unprovoked: normal (0), minor changes (1), less mobile and isolated (2), restless or very still (3); behavior-provoked: responsive and alert (0), unresponsive and not alert (3); (iii) clinical signs: normal respiratory rate (0), slight changes (1), decreased rate with abdominal breathing (2), marked abdominal breathing and cyanosis (3); (iv) hydration status: normal (0), dehydrated (5). One-time evaluation was conducted at 24 h after CLP, with two evaluators scoring separately and taking the average of each indicator. The higher score indicates worse clinical situation of the animal.

### 2.2 Serum and colon tissues collection

After measuring the body temperature and clinical score, five mice in each group were sacrificed and blood samples and colon tissues were collected. The blood was collected into a tube free of anticoagulants and placed on the laboratory bench at room temperature for 30 min, then centrifuged at 3,000×*g* for 5 min under 4°C, carefully transferred the serum to a clean tube, and then stored at −80°C as soon as possible. The serums TNF-α, IL-1β, and IL-6 levels were subsequently detected using enzyme-linked immunosorbent assay. Colon tissues were collected and divided into two parts, one of which was stored in −80°C as soon as possible and used for protein expression determination, the other part was fixed in 4% paraformaldehyde (PFA) for histological analysis.

### 2.3 GI motility and permeability test

Based on previous studies [19,20], FD4 (#60842-46-8-FD4-1 G, Sigma-Aldrich, USA) was used to evaluate GI mobility and permeability. Briefly, three mice in each group were gavaged 100 μL of 50 mg/mL FD4 dissolved in NC, at 24 h after CLP. Three hours later, the mice were anesthetized. Portal vein blood was collected and immediately centrifuged at 8,000×*g* for 10 min under 4°C to obtain the supernatant. The supernatant FD4 concentration was then quantified with a standard curve using a Multiskan Mk3 microplate reader (Thermo, USA) at an excitation wavelength of 488 nm. At the same time, the whole GI tract from stomach to colon was removed and imaged with Caliper IVIS Lumina III (PerkinElmer, USA). To quantify GI motility, the whole GI tract was divided into 12 segments and flushed with distilled water. The fluorescence of the purified recovered flushing solution was measured using the fluorescence microplate reader, and then fluorescence percentage of flushing solution of each segment was calculated.

### 2.4 Enzyme-linked immunosorbent assay

The levels of serum IL-1β, IL-6, and TNF-α were measured using commercial ELISA kits according to the instructions of the manufacturer (China). In brief, 50 μL of sample (10 μL serum diluted with 40 μL sample dilution buffer) and 100 μL of horseradish peroxidise (HRP)-conjugated detection antibodies were successively added into each well. The plate was incubated for 1 h at 37°C and washed five times. The substrate was then added and incubated in the dark at 37°C for 15 min. Then 50 μL stop solution was added to stop the enzyme reaction and absorbance was measured at 450 nm. The concentration of inflammatory factors was calculated using the standard curve.

### 2.5 Histological analysis

The PFA-fixed colon tissues were dehydrated and embedded in paraffin, and then sectioned at 5 μm thickness. Sections were stained with hematoxylin and eosin (H&E) for histological observation to examine the pathology of colon injury. Immunohistochemical staining of 5-HT was performed to evaluate the effect of PT treatment on 5-HT production. Alcian blue and periodic acid-Schiff (AB-PAS) staining was used to observe the changes of mature goblet cells. The stained slices were examined under the light microscope, and the images were captured (three random ×200 magnified fields in one section per mouse, five mice per group). The images were then analyzed using the ImageJ software.

### 2.6 RNA isolation and real-time PCR analysis

RNA extraction and cDNA synthesis were carried out according to the manufacturer’s instructions. Total RNA in the colon tissues was isolated using RNAiso Plus reagent (TaKaRa Biotechnology Dalian Co. Ltd, Dalian, China) and reverse transcribed into cDNA using PrimeScript RT Master Mix (TaKaRa). Primers for each gene were designed using NCBI Primer-BLAST and were synthesized by Sangon Biotech (Shanghai, China). The primers were as follows: occludin, 5′-ATCTTGGGAGCCTGGACATT-3′ (forward) and 5′-CCTCTGTCCCAAGCAAGTGT-3′ (reverse); claudin-1, 5′-AAAGCACCGGGCAGATACAG-3′ (forward) and 5′-GGGGGTCAAGGGGTCATAGA-3′ (reverse); zonula occludens-1 (ZO-1), 5′-CTCTCCTGTACCTCTTGAGCC-3′ (forward) and 5′-CAGAAATCGTGCTGATGTGCC-3′ (reverse); mucins (Muc)-2, 5′-GCCCACCTCACAAGCAGTAT-3′ (forward) and 5′-GTCATAGCCAGGGGCAAACT-3′ (reverse); tryptophan hydroxylase 1 (TPH1), 5′-TAAAGCAGTCTTGCCTGGTCAC-3′ (forward) and 5′-GAGGCCCGTGGACATACTTCT-3′ (reverse); TPH2, 5′-CCAGTCGGTGAGTTGTGGAA-3′ (forward) and 5′-CTGTGATGCAAAGCGTGGAG-3′ (reverse); monoamine oxidase A (MAO-A), 5′-ACTTACCCATTCCGTGGTGC-3′ (forward) and 5′-ACCACAGGGCAGATACCTCA-3′ (reverse); serotonin reuptake transporter (SERT), 5′-GATCAGCACTCCAGGGACAC-3′ (forward) and 5′-TCAGAGATGAGGAGTCGGGG-3′ (reverse); β-actin, 5′-GCTTCTAGGCGGACTGTTACT-3′ (forward) and 5′-GCCTTCACCGTTCCAGTTTTT-3′ (reverse). RT-PCR was performed with 2 μL cDNA and SYBR Premix Ex Taq (TaKaRa) on ABI 7500 system (ABI, California, USA). PCR conditions: 40 cycles, 95°C for 30 s, 60°C for 34 s, and 72°C for 30 s. β-Actin was used as an internal control to normalize gene expression. The relative expression level of candidate genes was analyzed using the 2^−ΔΔCt^ method.

### 2.7 Western blot analysis

Western blot was performed as previously described with modifications [21]. Briefly, the colon tissues were homogenized in cold RIPA lysis buffer, then centrifuged at 14,000×*g* for 15 min under 4°C to collect the supernatant. The protein concentration was quantified using BCA protein assay kit. The same amount of protein from each sample was dissolved in the loading buffer, denatured at 95°C for 5 min, and separated on 10% SDS-PAGE mini gels, then transferred to PVDF membranes. The membranes were blocked in 5% skimmed milk/Tris-buffered saline with 0.5‰ Tween-20 for 1 h at room temperature, then probed with primary antibodies, including β-actin (1:5,000, #3700; Cell Signaling Technology [CST], USA), p65 (1:1,000, #8242; CST), phospho-p65 (1:1,000, #3031; CST), p38 (1:1,000, #8690; CST), phospho-p38 (1:1,000, #4511; CST), p44/42 MAPK (Erk1/2) (1:1,000, #4695; CST), and phospho-p44/42 MAPK (Erk1/2) (1:1,000, #4370; CST). After incubation with primary antibodies overnight at 4°C, the membranes were blotted with the corresponding HRP-conjugated secondary antibodies (anti-rabbit IgG or anti-mouse IgG). The signals were detected by Western Blotting Luminol Reagent (sc-2048; Santa Cruz Biotechnology, CA, USA). The membranes were imaged with BioRad ChemiDoc^TM^ XRS+ System. Quantity One software (BioRad, CA, USA) was used to quantify the band intensity. The relative expression level of protein was expressed as the value of density ratio (target protein to β-actin in the same sample).

### 2.8 Statistical analysis

The data are expressed as mean ± SD. One-way ANOVA was used to compare the inflammatory factors, proteins, and histological variables among different groups. The survival study was conducted by log-rank test. GraphPad Prism 8 (GraphPad Software, Inc., La Jolla, CA, USA) was used for statistical analysis. *p* < 0.05 was considered statistically significant.

## 3 Results

### 3.1 Long-term supplementation of PT before CLP reduces mortality and inflammatory response in septic mice

**Figure 1:**
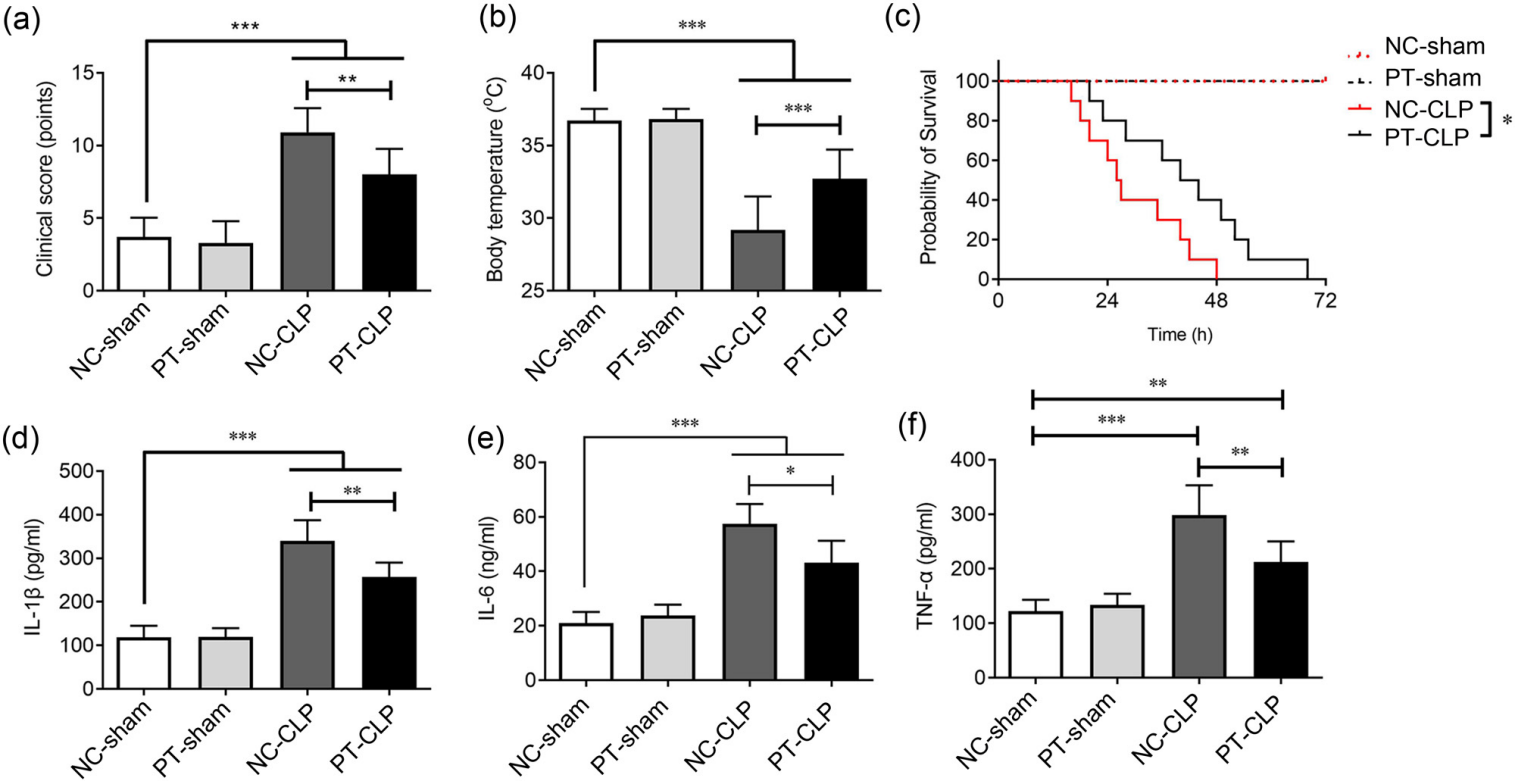
Supplementation with probiotics alleviates inflammatory reaction and increases survival of mice after CLP. (a) Clinical score: the clinical score of each animal was assessed at 24 h after CLP. The higher score indicates worse clinical situation of the animal (n: NC-sham or PT-sham = 10, NC-CLP = 8, PT-CLP = 9, mice that died before the evaluation were excluded). (b) Body temperature: the temperature of each animal was measured by rectal insertion of a temperature sensor while the mouse was under anesthesia at 24 h after CLP (n: NC-sham or PT-sham = 10, NC-CLP = 8, PT-CLP = 9, mice that died before the evaluation were excluded). (c) Survival curve: the mice were monitored to assess mortality for 120 h after CLP (*n* = 10/group). (d–f) Levels of IL-1β, IL-6, and TNF-α in serum were measured at 24 h after CLP (*n* = 5/group). The data represent mean ± SD, *p* < 0.05 was set as the threshold for significance. *p < 0.05, **p < 0.01, ***p < 0.001.

Mouse sepsis model was established to explore the preventive effect of long-term supplementation of PT on sepsis. After 4 weeks of PT gavage, the mice underwent CLP operation, and then the clinical score, body temperature, survival rate, and serum inflammatory cytokine level were measured. Compared with NC-CLP mice, PT-CLP mice had lower clinical scores (Figure 1a), better body temperature and close to normal body temperature (Figure 1b). The survival rate of PT-CLP mice was significantly increased (Figure 1c), but all animals died within 72 h after CLP. Inflammatory hallmark cytokines IL-1β, IL-6, and TNF-α constitute the cytokine storm during sepsis. ELISA analysis showed that PT pretreatment obviously reduced the levels of these three inflammatory cytokines (Figure 1d–f). This suggests that long-term oral pre-administration with PT has a preventive effect on CLP-induced sepsis in mice.

### 3.2 PT pre-administration improves GI peristalsis and permeability of septic mice

**Figure 2:**
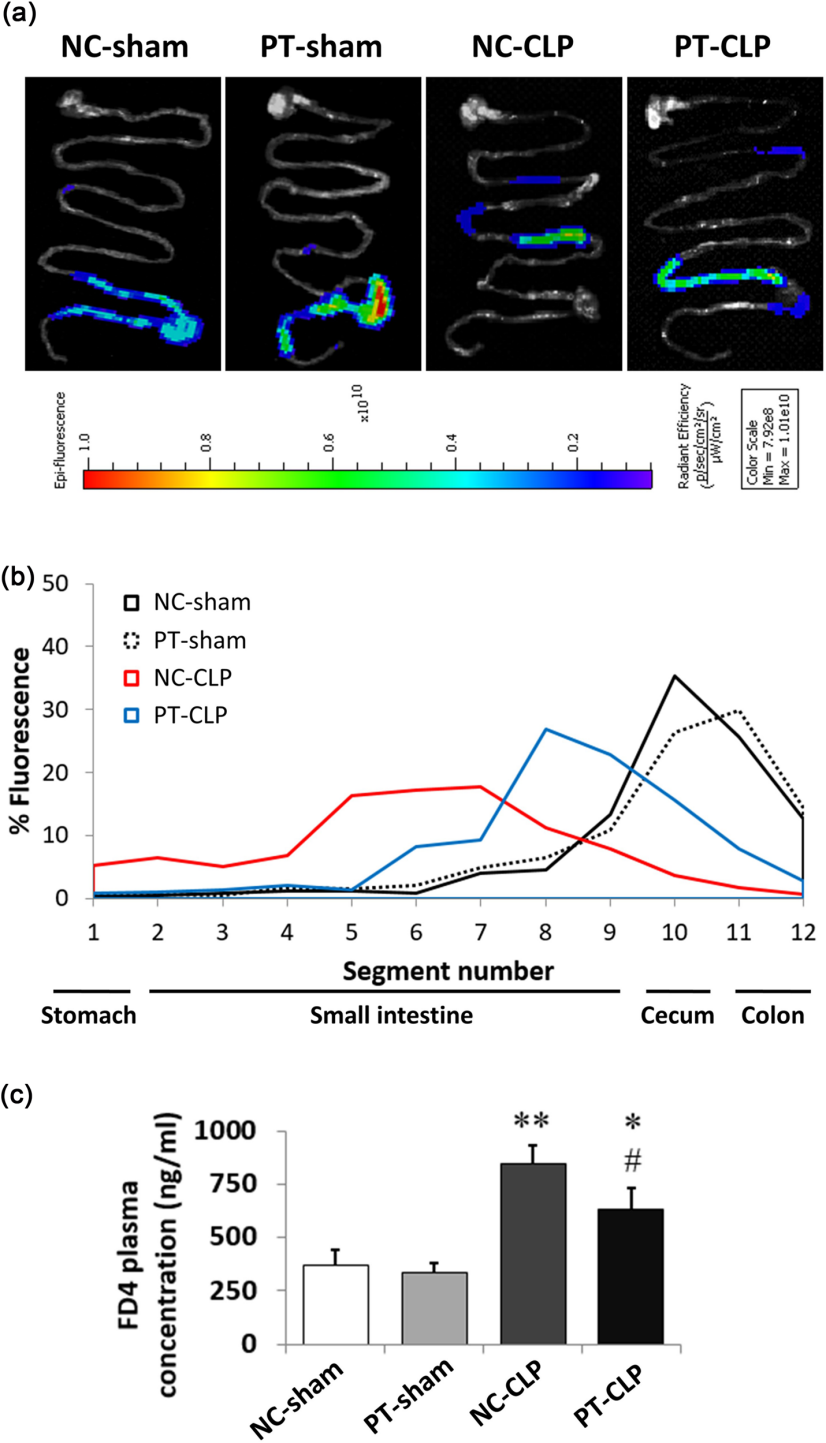
Supplementation with probiotics improves GI peristalsis and permeability of septic mice at 24 h after CLP. (a) Representative images: after intragastric administration of FD-4 in mice for 3 h, the whole GI tract from stomach to colon was taken and imaged with caliper IVIS lumina III. (b) Quantitative analysis showing the distribution of FD-4 in GI segments: the whole GI tract was divided into 12 segments and flushed with distilled water. The fluorescence of the recovered flushing solution was measured using a Multiskan Mk3 microplate reader at an excitation wavelength of 488 nm, and then fluorescence percentage of flushing solution of each segment was calculated. (c) Concentration of FD-4 in blood: the serum FD4 concentration was quantified with a standard curve using the fluorescence microplate reader. The data represent mean ± SD, *p* < 0.05 was set as the threshold for significance, *n* = 3/group. *p < 0.05, **p < 0.01 compared to the NC-sham group; ^#^ *p* < 0.05 compared to the NC-CLP group.

Sepsis has been shown to cause impaired GI motility. So, we first performed GI motility test after CLP operation to analyze the protective effect of PT on GI function. The representative images of different groups are shown in Figure 2a. Overall, after intragastric administration of fluorescein isothiocyanate-conjugated dextran (FD4) to mice for 3 h, mice in NC-sham and PT-sham groups showed high fluorescence intensity in cecum and colon, while FD4 mainly remained in the upper intestine of NC-CLP mice, indicating that sepsis weakened the GI peristalsis. Through probiotic pretreatment, the GI peristalsis of PT-CLP mice was enhanced, which showed that the fluorescence intensity from stomach to upper intestine was weak, while it was strong in the lower intestine segment (Figure 2a and b). Further detection of FD4 plasma concentration proved that PT pretreatment could reduce the high permeability caused by sepsis. The portal blood of NC-CLP mice contained higher levels of FD4, which was a sign of increased intestinal permeability after CLP surgery, while the concentration of FD4 in portal blood of PT-CLP mice decreased significantly (Figure 2c).

### 3.3 PT pre-administration protects GI barrier function of septic mice

**Figure 3:**
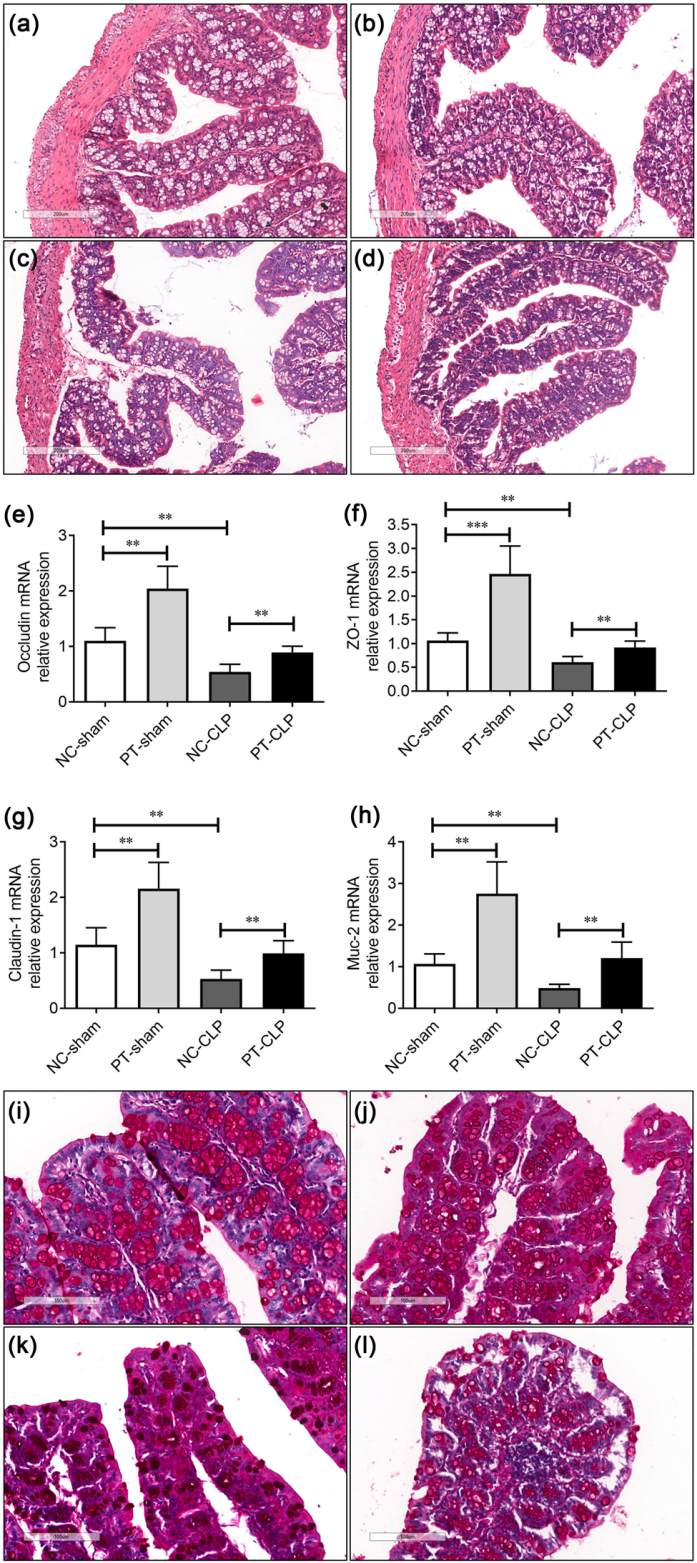
Probiotics supplementation protects GI barrier function of septic mice at 24 h after CLP. (a–d) Hematoxylin and eosin (e–h) staining of colon sections 24 h after CLP (representative images of *n* = 5/group are shown). (e–h) mRNA levels of tight junction proteins (occludin, ZO-1, and claudin-1) and Muc2 in colon were measured by real-time PCR (*n* = 5/group). (i–l) AB-PAS staining of mature goblet cells in colon sections (*n* = 5/group). a/i: NC-sham group, b/j: PT-sham group, c/k: NC-CLP group, d/l: PT-CLP group. Scale bar, 100 μm. The data represent mean ± SD, *p* < 0.05 was set as the threshold for significance. **p < 0.01, ***p < 0.001.

The decrease of GI motility may be caused by the injury of GI barrier. We further analyzed whether oral administration of PT could improve the GI barrier function after CLP operation. H&E staining showed that the colonic epithelium of NC-CLP mice was separated from the basement membrane, and the villi were also damaged, while the situation of PT-CLP mice was ameliorated, and the appearance of colon epithelium seemed regular (Figure 3a–d). Tight junctions are intercellular adhesion complexes that are critical to the barrier function of the epithelium. Dysregulated expression or dysfunction of tight junction proteins may lead to the destruction of tight junction integrity [22–24]. The mRNA levels of three tight junction proteins (occludin, ZO-1, and claudin-5) were detected. The results showed that their expression was lower in NC-CLP mice, indicating that the physical barrier of colon was destroyed, while probiotic pretreatment up-regulated the expression of these proteins (Figure 3e–g). In addition, mature intestinal goblet cells produce Muc, which is composed of the inner and outer layers of mucus, forming a protective mucus layer in the lumen [25]. The expression of Muc2 was significantly reduced in NC-CLP mice, but was upregulated by PT gavage (Figure 3h). AB-PAS histochemical staining showed that the number of mature goblet cells in the colon of NC-CLP mice decreased, while probiotic pretreatment before CLP maintained a high number (Figure 3i–l). These suggest that PT supplementation may protect GI barrier function of septic mice.

### 3.4 PT pre-administration upregulates 5-HT degradation pathway in the GI of septic mice

**Figure 4:**
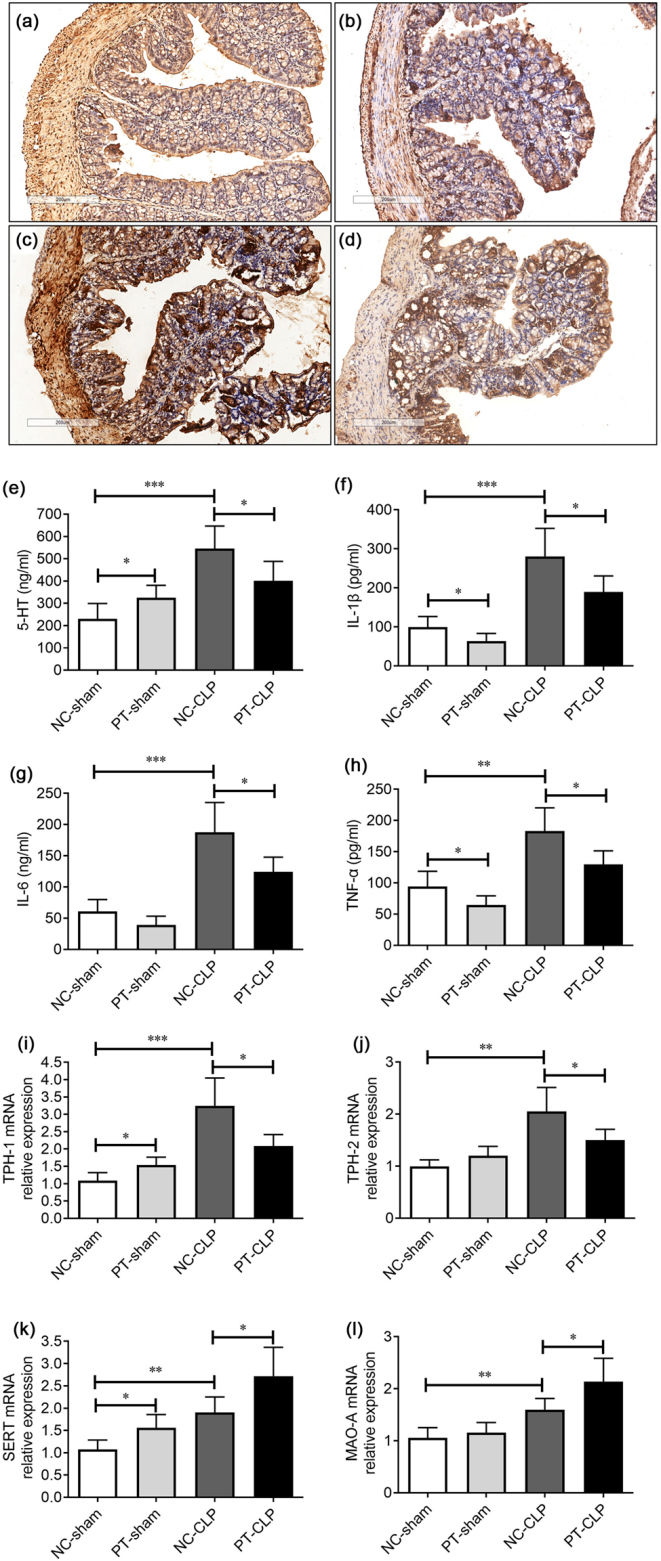
Probiotics supplementation upregulates 5-HT degradation pathway in the GI of septic mice. (a–d) Immunohistochemical staining of 5-HT in colon sections 24 h after CLP (representative images of *n* = 5/group are shown): (a) NC-sham group, (b) PT-sham group, (c) NC-CLP group, and (d) PT-CLP group. (e–h) ELISA analysis of 5-HT and the inflammatory cytokines (IL-1β, IL-6, and TNF-α) in colon tissues (*n* = 5/group). (i–l) mRNA expression levels of 5-HT reuptake transporter (SERT), synthases (THP-1, THP-2), and degradation enzyme (MAO-A) were detected by real-time PCR (*n* = 5/group). The data represent mean ± SD, *p* < 0.05 was set as the threshold for significance. *p < 0.05, **p < 0.01, ***p < 0.001.

During sepsis, the dysregulation of 5-HT induced by microflora ecological imbalance may promote inflammation, damage the intestinal mucosal barrier, facilitate bacterial translocation, and increase the mortality of sepsis [14]. PT intervention can reduce the high serotonin state [15]. Accordingly, the level of 5-HT in the colon of NC-CLP mice was remarkably increased after CLP operation, which was reduced by PT supplementation (Figure 4a–e). In addition, IL-1β, IL-6, and TNF-α were also significantly raised in the gut of NC-CLP mice, indicating the disturbed immune state, while the expression of these inflammatory cytokines was notably reduced in mice pre-fed with PT (Figure 4f–h).

However, the mechanism by which PT regulate 5-HT is not fully understood. 5-HT in the GI tract is mainly synthesized by intestinal chromaffin cells through the rate limiting enzyme TPH1 and released under mechanical and chemical stimulation of the intestinal cavity [26]. The termination of 5-HT signaling is almost dependent on the intracellular transport of selective SERT, which is expressed in all intestinal epithelial cells [27,28]. MAO-A is mainly responsible for 5-HT degradation in colon tissues [29]. Therefore, we tested whether probiotic pretreatment could affect the level of 5-HT by regulating the expression of THPs, SERT, and MAO-A. The mRNA levels of these key factors in the colon tissues were detected by real-time PCR. The results showed that the expression of THP-1 and THP-2 increased significantly in NC-CLP mice, which was decreased in PT-CLP mice pretreated with PT. While SERT and MAO-A of NC-CLP mice increased slightly, it remarkably increased in PT-CLP mice after administration of PT (Figure 4i–l). These evidence suggest that long-term supplementation with PT before CLP can inhibit 5-HT synthesis and promote 5-HT clearance in the gut of septic mice.

### 3.5 PT pre-administration inhibits NF-κB pathway, while activates ERK pathway

**Figure 5:**
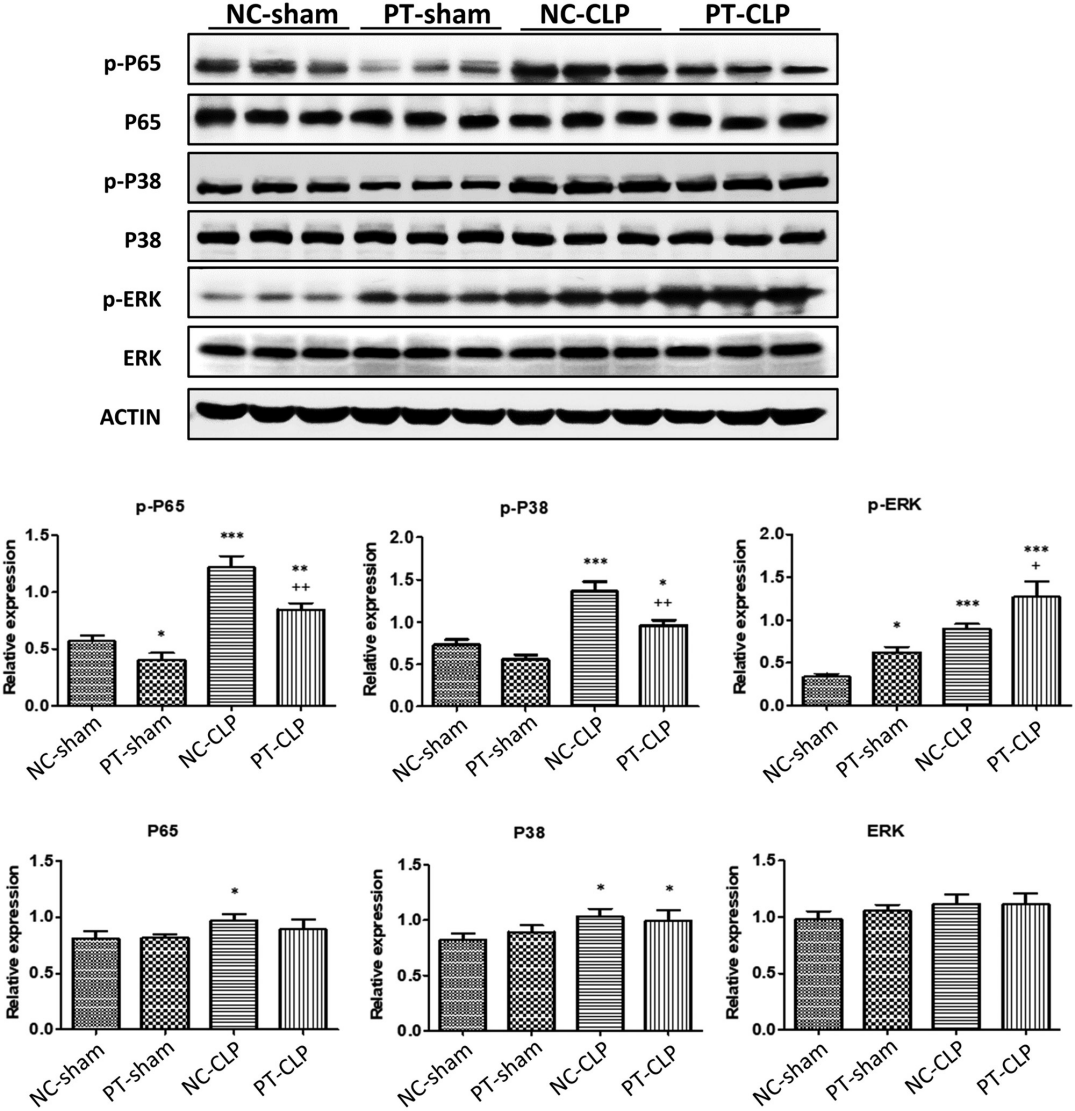
Probiotics supplementation inhibits NF-κB pathway, while activates ERK pathway. The expression levels of p-p65, p65, p-p38, p38, p-ERK, and ERK in colon tissues were detected by western blotting (upper panel, *n* = 3/group). Using actin as the internal reference, the density of related proteins was analyzed (lower panel). The data represent mean ± SD, *p* < 0.05 was set as the threshold for significance. *p < 0.05, **p < 0.01, ***p < 0.001.

The above experiments show that PT supplementation can reduce the inflammatory response caused by sepsis. It is well known that NF-κB is responsible for the early activation of inflammatory genes and is a key promoter of pathological processes such as inflammation, autoimmune diseases, and septic shock [30]. Therefore, we analyzed the effect of PT supplementation on NF-κB activity, and also tested its effect on p38 and ERK activity *in vivo*. We found that sepsis significantly activated NF-κB, p38, and ERK, as evidenced by their notably increased phosphorylation levels after CLP operation, while PT supplementation inhibited NF-κB and p38, but further activated ERK (Figure 5). It is reported that activation of NF-κB can promote the expression of THPs, suggesting that PT supplementation downregulated THPs expression through inhibiting NF-κB signal. Moreover, enhancing the activity of ERK by supplementing PT may be one of the reasons for the high level expression of SERT in the gut. These data suggest the multiple regulatory effects of PT supplementation on sepsis.

## 4 Discussion

During sepsis, the dysregulation of 5-HT may promote inflammation, disrupt the intestinal mucosal barrier, facilitate bacterial translocation, and increase the mortality of sepsis [14]. At the same time, the gut microbiota also participates in the regulation of 5-HT production [13]. Oral administration of PT can alter the gut microflora and downregulate 5-HT [15,31,32], thus being considered as an adjuvant therapy to reduce bacterial translocation and prevent sepsis [6,7]. However, the underlying mechanism is not entirely clear. In the present study, we pre-fed mice with PT for 4 weeks and then established a CLP mouse sepsis model to detect the effect of PT supplementation on sepsis-induced GI mucosal barrier dysfunction and explore the underlying mechanism, revealing the preventive effect of PT pre-administration on GI mucosal barrier during sepsis, and its mechanism may be partially mediated by activating the ERK signaling to upregulate the 5-HT degradation pathway.

The gut microbiome has been proven to regulate many homeostatic mechanisms of the host, including immune function and gut barrier protection [33]. Abnormalities in the gut microbiota will enable the expansion of intestinal pathogens, generate a strong pro-inflammatory response in the immune system, and reduce the production of beneficial microbial products such as short chain fatty acids, making the host susceptible to sepsis and having a negative impact on the outcome of sepsis [6,33,34]. Long-term administration of PT can maintain a balance of gut microbiota and reduce the risk of sepsis. Chen et al. demonstrated that LGG pretreatment improved the richness and diversity of intestinal microbiota in mice, reversing the imbalance of microbiota caused by sepsis, e.g., reducing conditional pathogenic bacteria, LPS producing bacteria, and facultative anaerobic bacteria, while increasing the abundance of bacteria related to energy acquisition and colon barrier recovery bacteria [4,6]. Based on this, in our study, long-term high concentration probiotic pretreatment may also first alter the structure of the mouse GI microbiota, thereby improving the clinical, histological, and molecular parameters of the GI mucosa of PT-CLP mice before CLP, making it different from NC-CLP mice and becoming the physiological basis for the subsequent formation of multiple pathways to defense against sepsis.

The CLP model of mouse sepsis can simulate the nature and evolution of severe sepsis in humans, and is currently considered the gold standard for sepsis research and the most widely used model for experimental sepsis [35–40]. In the process of sepsis, cytokines such as TNF-α, IL-1β, and IL-6 mediated inflammatory response plays an important role [37], which may lead to endothelial and epithelial damage, affect vascular permeability and heart function, and eventually result in tissue necrosis and organ failure [4]. In this study, we found that PT pretreatment significantly reduced the levels of serum TNF-α, IL-1β, and IL-6 induced by sepsis. Accordingly, the activity of NF-κB, one of the key inflammatory promoters responsible for the early activation of these inflammatory genes [30], was remarkably downregulated by PT pretreatment. Meanwhile, PT pretreatment also ameliorated colonic pathological damage, enhanced GI peristalsis, and reduced high permeability caused by sepsis. Our findings indicated that PT pretreatment can alleviate inflammation and maintain the integrity of GI epithelium to prevent GI mucosal barrier dysfunction in sepsis.

Tight junctions are intercellular adhesion complexes that are crucial for the barrier function of the epithelium. The expression imbalance or dysfunction of tight junction proteins may lead to the destruction of tight junction integrity [22–24]. Yoseph et al. measured the expression of tight junction proteins including claudins 1, 2, 3, 4, 5, 7, 8, 13, and 15, JAM-A, occludin, and ZO-1 in mice after CLP to determine the potential mechanisms of increased intestinal permeability. They found that occludin and claudin-5 decreased due to sepsis [41]. Tan et al. reported that CLP mice exhibited severe intestinal damage, with significantly reduced levels of occludin and ZO-1 [42]. A recent study showed that the intestinal barrier of mice with ulcerative colitis induced by sodium dextran sulfate was severely damaged. Treatment with *Scutellaria baicalensis* polysaccharides significantly increased the abundance of Firmicutes, Bifidobacteria, Lactobacillus, and Rosaceae in the intestine, and upregulated the expression of occludin, claudin-5, and ZO-1 to repair the intestinal barrier [43]. In our study, the expressions of occludin, ZO-1, and claudin-5 were reduced in NC-CLP mice, indicating that the physical barrier of intestine was disrupted, while PT pretreatment upregulated these proteins in PT-CLP mice. In addition, mature intestinal goblet cells produce Muc, which is composed of inner and outer layers of mucus, forming a protective mucus layer in the lumen [25]. The expression of Muc2 was significantly reduced in NC-CLP mice, but upregulated by long-term pretreatment with PT. These results suggest that PT supplementation may restore GI barrier function of septic mice by upregulating the tight junction proteins.

5-HT is an important signaling molecule in the GI tract. Under physiological conditions, 5-HT targets intestinal cells, smooth muscles, and intestinal neurons, mainly regulating GI motility, epithelial secretion, and vasodilation through 5-HT_3_ and 5-HT_4_ receptors [12,44]. However, 5-HT also exerts nonconventional roles under pathophysiological conditions. The increased availability of mucosal 5-HT may promote inflammation, reduce fecal excretion, delay upper GI transit, and increase permeability [44,45]. In this study, we demonstrated that pre-administration of PT can reduce the levels of 5-HT and inflammatory cytokines in the colon of septic mice.

5-HT in the GI tract is mainly synthesized by the rate limiting enzyme TPH1 in intestinal chromaffin cells and released under mechanical and chemical stimulation in the intestinal cavity [26]. The termination of 5-HT signaling is almost dependent on the intracellular transport of SERT, which is expressed in all intestinal epithelial cells [27,28]. However, MAO-A is mainly responsible for the degradation of 5-HT in colon tissues [29]. Many studies have shown that the decrease of intestinal SERT level in IBS-D patients is related to the increase of serotonin and the deterioration of symptoms [46]. It is also reported that SERT expression in colon biopsy specimens of IBS-D patients is significantly reduced [47]. We found that the expression of THP-1 and THP-2 increased significantly in NC-CLP mice, which was decreased in PT-CLP mice pretreated with PT. While SERT and MAO-A of NC-CLP mice increased slightly, it increased remarkably in PT-CLP mice after administration of PT. Further research revealed that NF-κB and ERK signals may be involved in the regulation of SERT and MAO-A expression by pre-feeding with PT. These evidence suggest that PT pretreatment can inhibit the synthesis of 5-HT in the intestine of septic mice and promote the clearance of 5-HT, thereby playing a preventive role in sepsis.

So far, increasing evidence indicate that improving the GI microbiota through long-term pre-feeding of PT can generate multiple pathways to protect the GI mucosa and prevent adverse reactions caused by sepsis. For example, (1) regulating the host’s immune response: oral administration of LGG can activate the IL-22BP/IL-22/STAT3 pathway in the ileum to improve inflammation [8], *Lactobacillus flexneri* WiKim38 induces IL-10 production from dendritic cells and alleviates mouse colitis [10], and *Lactobacillus carinii* SBT2055 induces TGF-β in dendritic cells and activation of TLR2 signaling to produce IgA in the small intestine [11]. (2) Regulating metabolic profile: LGG can reduce the mortality rate of septic mice by regulating the composition and metabolic profile of intestinal microbiota, especially the production of short-chain fatty acids, which are crucial for immune effects and epithelial cell function [6,33]. Therefore, the regulation of the 5-HT pathway revealed in this study is only one of the mechanisms by which PT improve GI function in septic mice. However, our research still has some limitations. For example, changes in gut microbiota after long-term administration of PT have not yet been detected, and further research is needed on the downstream pathways of 5-HT.

## 5 Conclusions

Based on the findings of this study, we conclude that the mechanism of PT pretreatment prevents GI mucosal barrier dysfunction in septic mice and may be partly through upregulation of 5-HT degradation pathway by inhibiting NF-κB signal and activating ERK signal.
